# Supplementary material for: The Habc domain of syntaxin 3 is a ubiquitin binding domain
Source: Sci Rep. 2020 Dec 7;10:21350. doi: 10.1038/s41598-020-78412-0 (PMC7721868; doi:10.1038/s41598-020-78412-0)
Supplement: Supplementary file 1 — Supplementary Informations. [file 41598_2020_78412_MOESM1_ESM.pdf]

The H_{abc} Domain of Syntaxin 3 is a Ubiquitin Binding Domain

Adrian J. Giovannone[1#], Elena Reales[1,2,#], Pallavi Bhattaram[1,3], Sirpi Nackeeran[1], Adam B. Monahan[1], Rashid Syed[1,4] and Thomas Weimbs[1*]

Author Affiliations:

[1]Department of Molecular, Cellular, and Developmental Biology; and Neuroscience Research Institute, University of California, Santa Barbara, California, USA.

Footnotes:

*Correspondence to: Thomas Weimbs, PhD, Department of Molecular, Cellular & Developmental Biology, University of California Santa Barbara, Santa Barbara, California 93106-9625, weimbs@ucsb.edu

[2]Present address: Department of Organic Chemistry, School of Sciences, University of Cadiz; and Biomedical Research and Innovation Institute of Cadiz (INiBICA), Spain.

[3]Present address: Department of Orthopaedics, Emory University School of Medicine, Atlanta 30322, USA.

[4]Present address: Department of Chemistry and Biochemistry, California State University Northridge, Northridge, CA 91330-8262

# Contributed equally/ Co-first authors

b

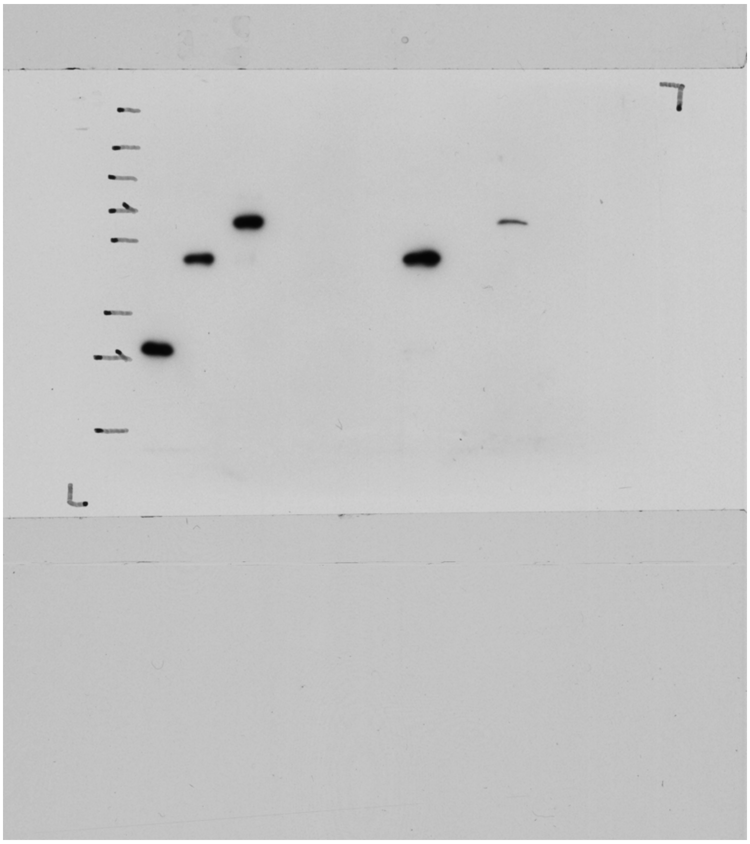

c

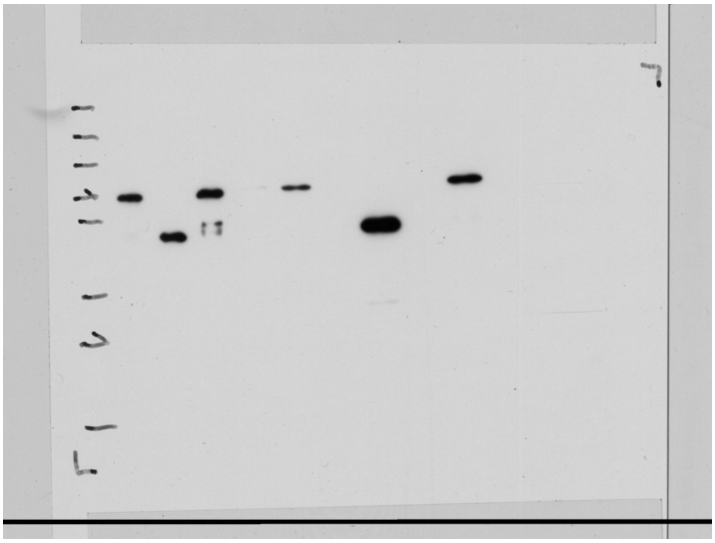

Figure 1-originals

a

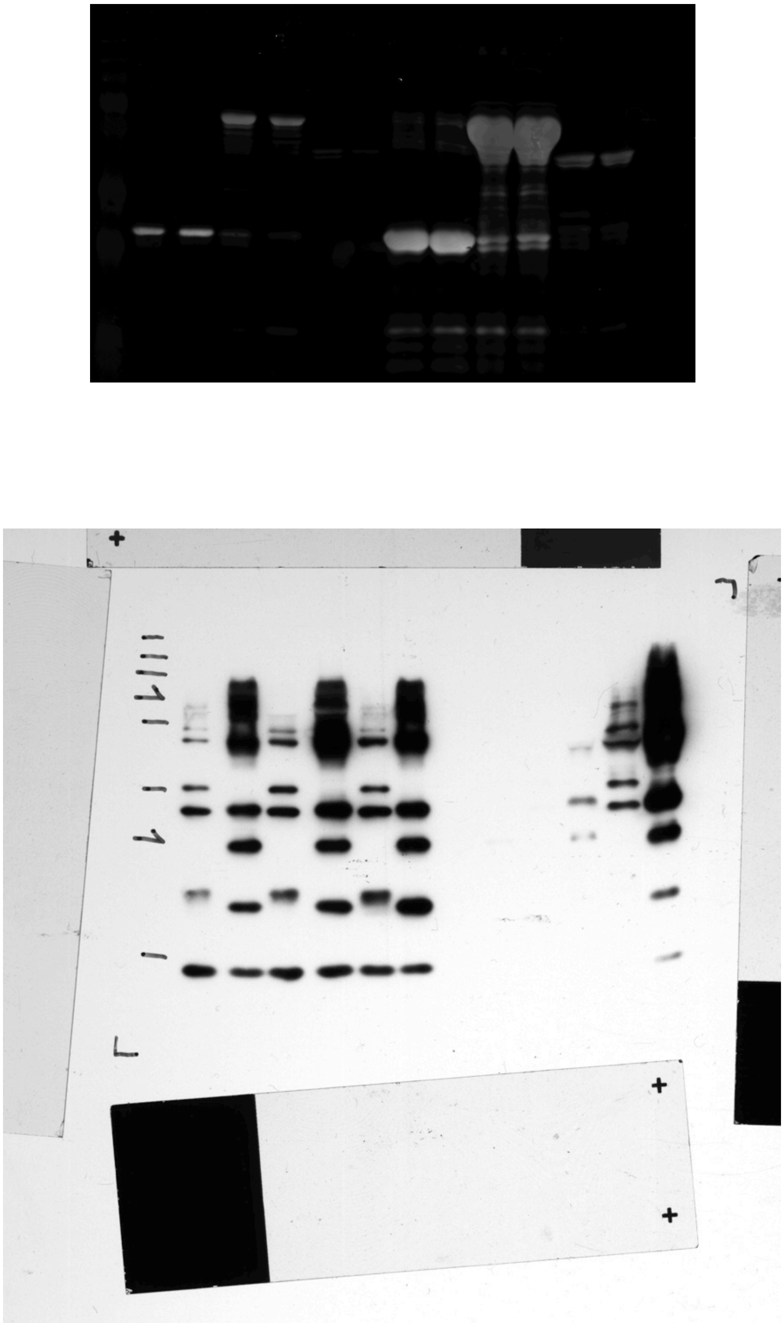

Figure 2a- originals

b

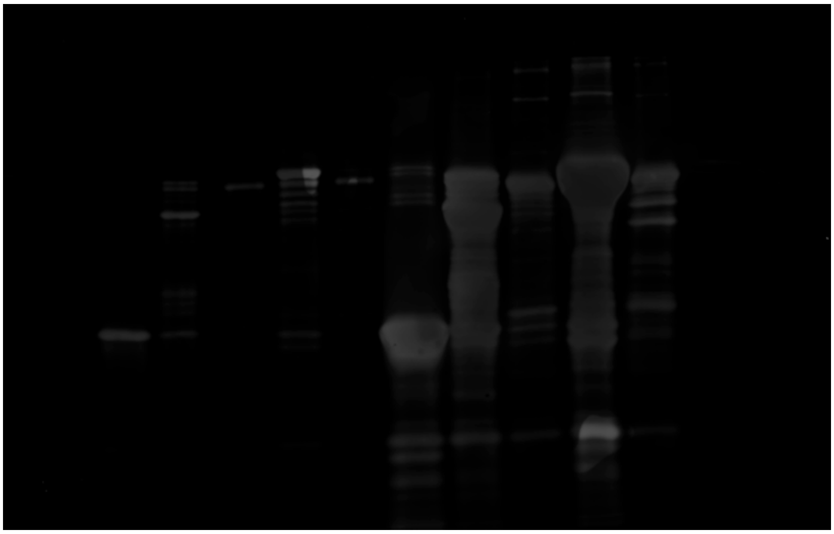

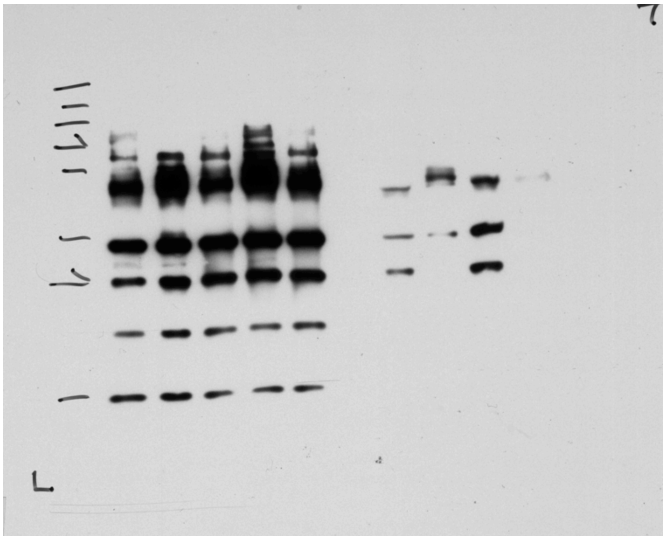

Figure 2b- originals

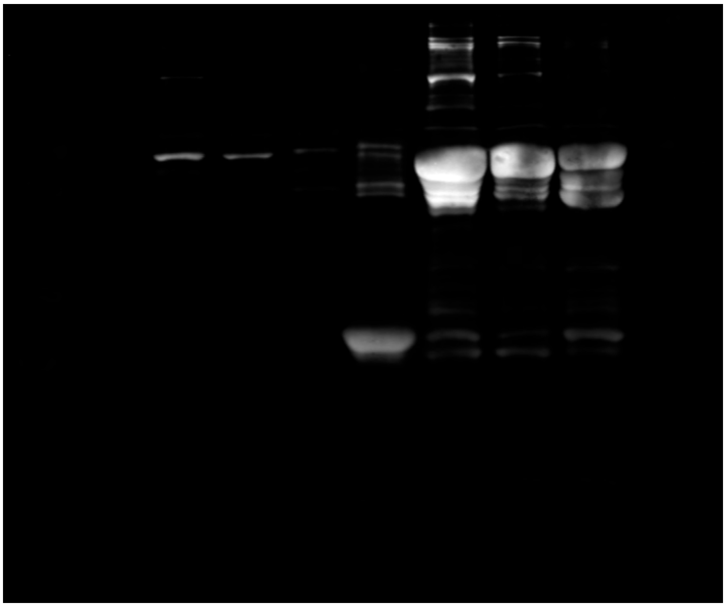

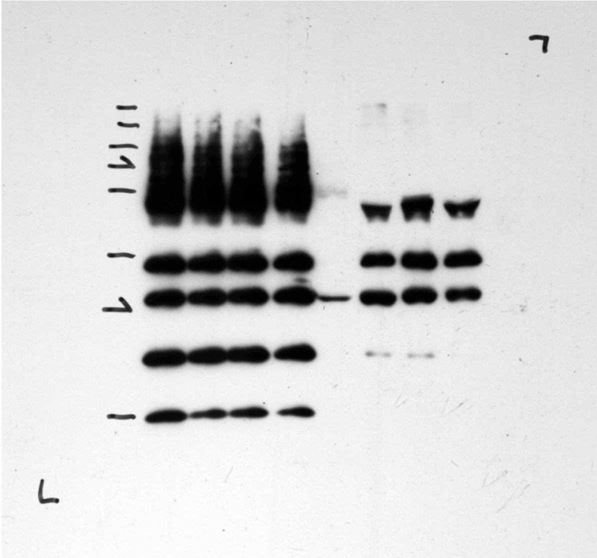

Figure 5- originals
